# Infiltrating mast cells enhance prostate cancer invasion *via* altering LncRNA-HOTAIR/PRC2-androgen receptor (AR)-MMP9 signals and increased stem/progenitor cell population

**DOI:** 10.18632/oncotarget.3651

**Published:** 2015-03-26

**Authors:** Lei Li, Qiang Dang, Hongjun Xie, Zhao Yang, Dalin He, Liang Liang, Wenbing Song, Shuyuan Yeh, Chawnshang Chang

**Affiliations:** ^1^ Department of Urology, Sex Hormone Research Center, The First Affiliated Hospital, Xi'an Jiaotong University, Xi'an, China; ^2^ Departments of Pathology and Urology, George Whipple Lab for Cancer Research, The Wilmot Cancer Center, University of Rochester Medical Center, Rochester, New York, USA; ^3^ Sex Hormone Research Center, China Medical University/Hospital, Taichung, Taiwan

**Keywords:** prostate cancer, mast cells, androgen receptor, stem cells, HOTAIR-PRC2

## Abstract

Early studies indicated that selective inflammatory immune cells in the prostate tumor microenvironment might be able to influence prostate cancer (PCa) progression. Here we found treating PCa cells with androgen deprivation therapy (ADT) results in the recruitment of more mast cells, which might then increase PCa cell invasion *via* down-regulation of AR signals in 4 different PCa cell lines. Mechanism dissection revealed infiltrating mast cells could decrease AR transcription *via* modulation of the PRC2 complex with LncRNA–HOTAIR at the AR 5′ promoter region in PCa cells. The consequences of suppressing AR may then increase PCa cell invasion *via* increased MMP9 expression and/or increased stem/progenitor cell population. The *in vivo* mouse model with orthotopically xenografted PCa CWR22Rv1 cells with/without mast cells also confirmed that infiltrating mast cells could increase PCa cell invasion *via* suppression of AR signals. Together, our results provide a new mechanism for the ADT-enhanced PCa metastasis *via* altering the infiltrating mast cells to modulate PCa AR-MMP9 signals and/or AR-stem/progenitor cell population. Targeting these newly identified inflammatory mast cells-AR signals may help us to better suppress PCa metastasis at the castration resistant stage.

## INTRODUCTION

Prostate cancer (PCa) is the second most commonly diagnosed cancer worldwide [1]. The androgen receptor (AR) signals play critical roles for PCa initiation and progression [2, 3] and androgen deprivation therapy (ADT) is the standard treatment for PCa at late stages, with better efficacy during the first 12-24 months before development into castration resistance [4-6].

A growing body of evidence suggests that several immune cells (including macrophages, T cells, neutrophils and mast cells) with their secreted inflammatory cytokines may play roles to influence the PCa progression [7-9]. Mast cells function in many conditions including allergies, angiogenesis, tissue remodeling, and immunomodulation of cancer [10, 11]. Mast cells were detected in the prostate tumor microenvironment (pTME) and their expression has been linked to the PCa progression [12]. There are two different types of mast cells in tumors, intra- and peri-tumoral mast cells. and they may play different roles in tumor progression [12]. The intra-tumoral mast cells play a protective role in tumor development yet the peri-tumoral mast cells play a negative role to promote tumor progression [12]. Most mast cells were detected around the peri-tumoral area in the pTME, and ADT with castration may increase the recruitment of mast cells to PCa [13]. However, the detailed mechanism how recruited mast cells may contribute to the PCa progression remains unclear.

Cancer stem/progenitor cells have been well-documented to play important roles in tumor initiation and metastasis [14, 15]. Earlier reports found that PCa also contained stem/progenitor cells that might play essential roles in prostate tumorigenicity and metastasis [16, 17] and AR might play differential roles in PCa stem/progenitor cells vs. PCa parental cells [18].

Polycomb Repressive Complex 2 (PRC2) is one of the two classes of the polycomb-group proteins (PcG), and this complex has histone methyltransferase activity and primarily trimethylate histone H3 on lysine 27 [19, 20], a mark of transcriptionally silent chromatin. It is believed that PRC2, comprised of H3K27 histone methyltransferase (HMTase) EZH2 and core components SUZ12 and EED, initiates this histone modification and that the subsequent Polycomb Repressive Complex 1 (PRC1) maintains this modification and promotes chromatin compaction [21].

The long non-coding RNA, HOTAIR (for HOX antisense intergenic RNA), is a human gene located on the chromosome 12 [22]. It is the first example of an RNA expressed on one chromosome that has been found to influence transcription on another chromosome. HOTAIR interacts with PRC2 [21] and is required for gene-silencing of the HOXD locus by PRC2 [23]. Higher expression of HOTAIR in primary breast tumors is a significant predictor of subsequent metastasis and death [24].

Here we found ADT treatment of PCa resulted in increased cell invasion *via* increased numbers of recruited mast cells-PCa AR-MMP9 signals and alteration of the AR-induced stem/progenitor cell population.

## RESULTS

### PCa cells treated with ADT using casodex or enzalutamide recruit more mast cells

**Figure 1:**
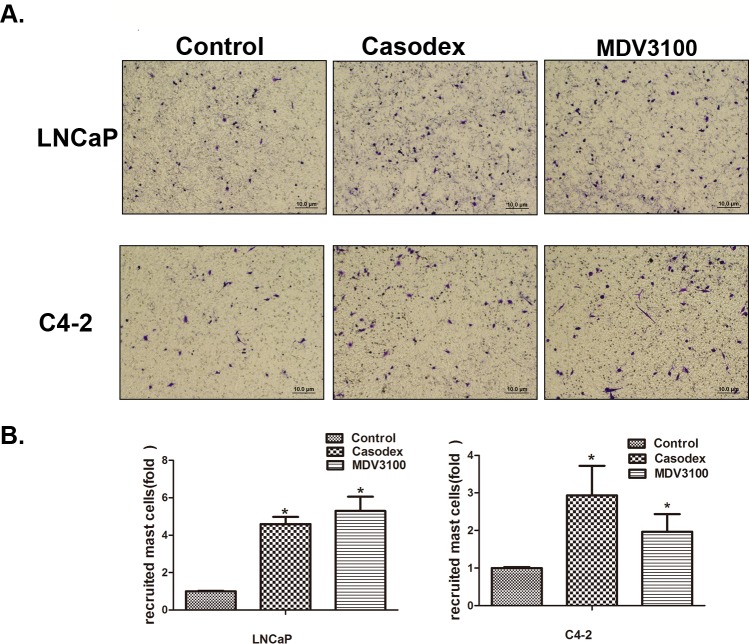
Prostate cancer cells recruit more mast cells after the treatment with casodex or enzalutamide (MDV3100) **A**. Mast cell recruitment capabilities were assayed using LNCaP and C4-2 cells conditioned media (CM) treated with 10 μM casodex or 10 μM MDV3100. **B**. Quantification data for mast cell migration. Results were presented as the mean ± SEM. Statistical analysis was done by two-tailed Student's t test, * p < 0.05.

Early studies suggested that mast cells could be recruited to various tumors cells, including PCa [12]. Here we applied the Boyden chamber migration system to assay the human mast cells (HMC-1) migration ability to LNCaP and C4-2 cells after treatment with 10 μM casodex or 10 μM enzalutamide, and the results revealed cells treated with both anti-androgens recruited more HMC-1 cells than DMSO control treated cells (Figure 1A and 1B).

### PCa cells have better capacity than normal prostate cells to recruit more mast cells

We applied IHC staining in the human PCa samples using the tryptase as a marker of mast cells, and found more mast cells were recruited to the PCa as compared to the adjacent normal prostate tissue (Figure S1A-B). To confirm these *in vivo* clinical data, we assayed the HMC-1 cells migration ability to PCa LNCaP cells *vs.* normal prostate RWPE1 cells by using the Boyden chamber migration system (Figure S1C), and the results showed LNCaP cells have better capacity to recruit more mast cells than normal prostate RWPE1 cells (Figure S1D-E). Similar results were obtained when we replaced LNCaP cells with other PCa cells, including C4-2, C4-2B and CWR22RV1 cells (Figure S1D-E).

Together, both *in vivo* human clinical data and *in vitro* cell co-culture data proved that PCa cells could recruit more mast cells than normal prostate cells.

### Increased infiltrating mast cells to PCa enhanced PCa cell invasion

**Figure 2:**
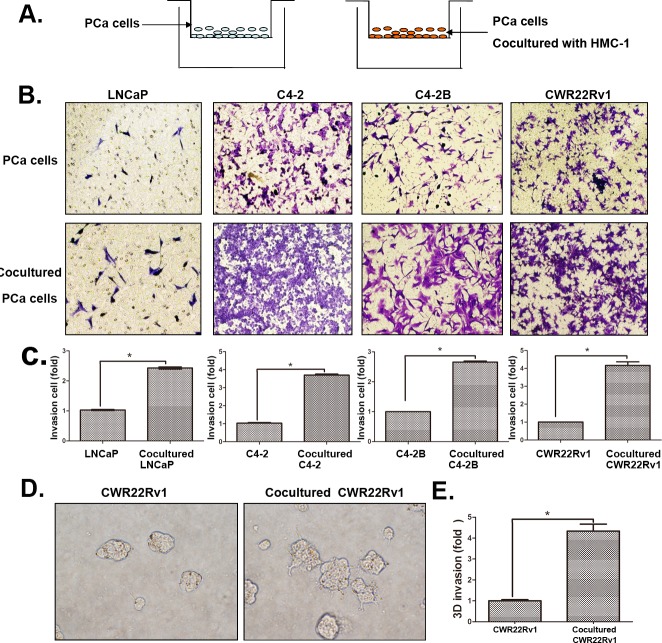
Increased infiltrating mast cells to PCa enhanced PCa cell invasion **A**. The cartoon illustrated the invasion assay. In brief, we co-cultured four different PCa cells with/without mast (HMC-1) cells for 2 days, and then washed out the HMC-1 cells. The co-cultured PCa cells were collected and re-seeded in the 8 μm pore size insert wells pre-coated with matrigel to perform invasion assays. **B**. Images show mast cells co-cultured PCa cells have a higher invasiveness. The top panels show untreated PCa cells as control, the bottom panels show PCa cells co-cultured with HMC-1 cells. **C**. Quantification data of changed PCa cells invasion. **D**. 3D invasion assay results showed mast cells co-cultured PCa cells have an increased invasiveness. **E**. Quantification data of 3D invasion. * p < 0.05.

We then applied chamber invasion assays in co-culture system (Figure 2A) to examine the consequences of increased infiltrating mast cells on PCa progression. We first treated HMC-1 cells with the differentiation reagent phorbol 12-myristate 13-acetate (PMA) to induce the mast cell differentiation and maturation. We then used these matured mast cells HMC-1 co-cultured with 4 different PCa cells (LNCaP, C4-2, C4-2B and CWR22RV1) for their capacity to invade (Figure 2B). As shown in Figure 2C-D, PCa (LNCaP, C4-2, C4-2B and CWR22RV1) cells with recruited mast cells all become more invasive in the Boyden chamber invasion system, suggesting that the recruitment of mast cells to PCa cells might increase their invasiveness.

### Mechanism dissection why recruited mast cells increased PCa cell invasion

**Figure 3:**
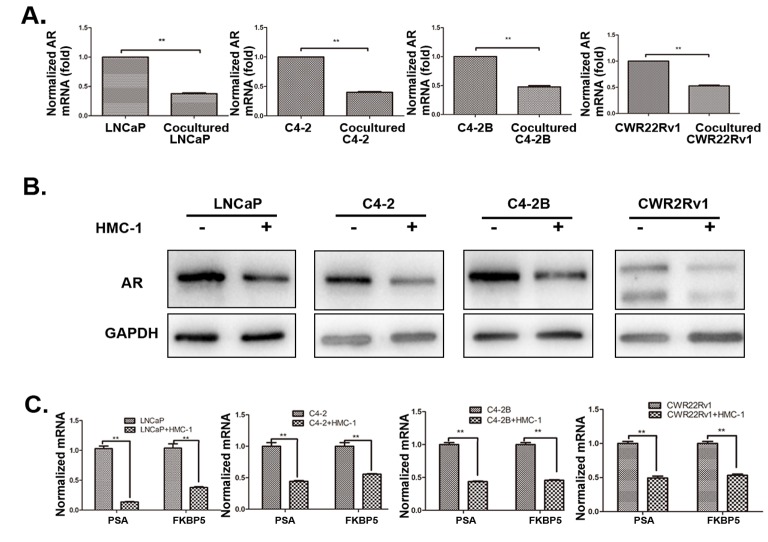
Recruited mast cells enhanced PCa cell invasion via alteration of AR signaling All the detailed co-culture conditions were described in Materials and Methods. **A**. AR mRNA level was down-regulated in PCa cells after co-cultured with HMC-1 cells. **B**. AR Protein level is down-regulated in PCa cells after co-cultured with mast cells. **C**. QPCR results confirmed AR target genes (PSA and FKBP5) were also down-regulated in PCa cells after co-cultured with mast cells. ** p < 0.01.

To dissect the molecular mechanisms why increased infiltrating mast cells could increase PCa cell invasion, we examined the AR expression since recent reports demonstrated targeting PCa AR (with siRNA) could increase PCa cell invasion [8, 25]. As shown in Figure 3A-B and S2 (for LNCaP and C4-2 cells), recruited HMC-1 cells or conditioned media (CM) after co-culture with PCa cells could decrease AR expression at both protein and mRNA levels in all four PCa cell lines (LNCaP, C4-2, C4-2B and CWR22RV1). We further confirmed these conclusions by demonstrating that co-culturing PCa cells with HMC-1 cells could also decrease the expression of AR downstream target genes including PSA and FKBP5 expression in all four PCa cell lines (Figure 3C), suggesting that more infiltrating mast cells to PCa could increase PCa cell invasion *via* down-regulation of AR signaling in PCa cells.

**Figure 4:**
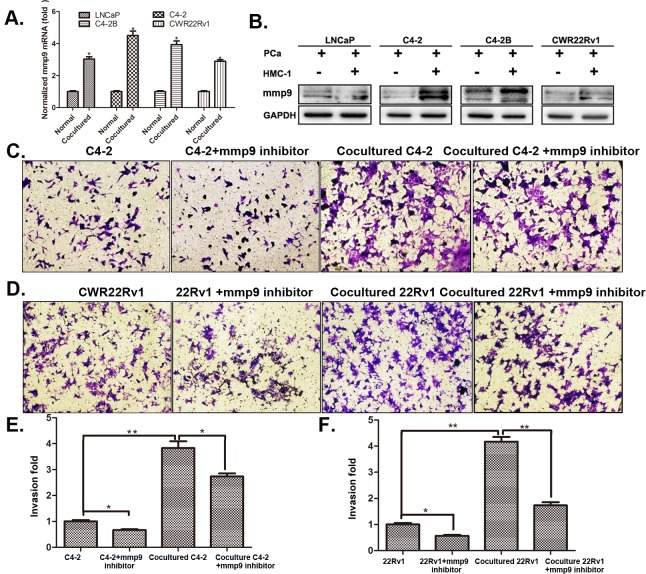
MMP9 expression in PCa cells after co-cultured with mast cells **A**. QPCR results of MMP9 (mmp9) expression in 4 different PCa cells after being co-cultured with HMC-1 cells. **B**. Western blot confirmed the increased MMP9 expression in PCa cells after being co-cultured with HMC-1 cells. C-D. Targeting MMP9 by specific inhibitor to interrupt mast cell mediated (**C**) C4-2 and (**D**) CWR22Rv1 (22Rv1) cells invasion. **E**. Quantification results of (**C**). F. Quantification results of (**D**). * p < 0.05, ** p < 0.01.

To further dissect the mechanism why infiltrated mast cells suppressed AR signaling could increase PCa cell invasion, we then applied focus-array with several PCa metastasis-related genes (Table S1) and found MMP9 was consistently increased in all four different PCa cell lines (with many other metastasis-related genes increased in one, two or three PCa cell lines, and qPCR and western blot assays confirmed the increased MMP9 at both mRNA (Figure 4A) and protein level (Figure 4B). Importantly, we also demonstrated that knocking-down AR increased MMP9 expression, yet suppression of MMP9 with the inhibitor failed to alter the expression of AR, suggesting MMP9 is a downstream target gene of AR (Figure 4C-D and S3A-B) in C4-2 and CCWR22Rv1 cells.

Together Figure 4 and S3 suggest that recruited mast cells may need to function through modulation of AR-MMP9 signals to increase PCa cell invasion.

### Recruited mast cells increased PCa cell invasion *via* modulation of AR signals to alter the CD133^+^ stem/progenitor cell population

**Figure 5:**
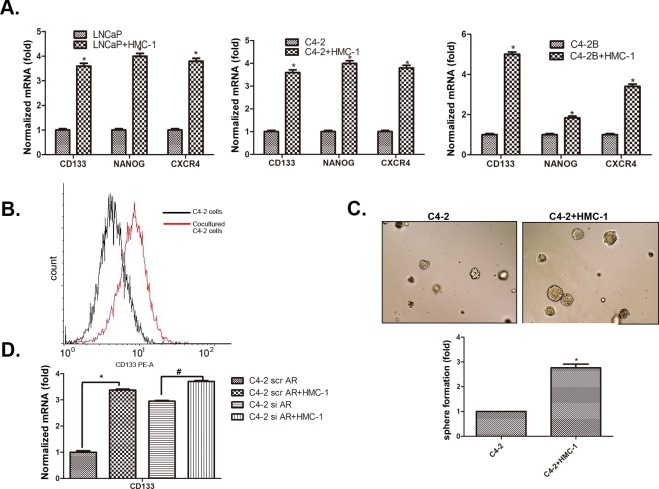
Recruited mast cells enhance PCa cell invasion *via* alteration of AR-mediated CD133 stem/progenitor cell population **A**. Co-culture of mast cells with PCa cells led to increased stem cell marker proteins, CD133, NANOG, and CXCR4. **B**. Flow cytometry analysis showed increased CD133^+^ cell population in PCa after being co-cultured with HMC-1 cells. **C**. Sphere formation assay validated the increased stem/progenitor cells. The PCa cells were co-cultured with or without HMC-1 cells for 5 days. PCa cells were then mixed with matrigel (1:1, v/v), plated in 24-well plates, and cultured for 7 days. Quantification results are presented in the lower panel. D. Knockdown of AR in PCa cells changes their responsiveness to mast cells-promoted CD133^+^ stem cell marker expression. * p < 0.05.

Early studies suggested that stem/progenitor cells have higher invasive capacity to promote tumor metastasis [26]. We were interested in whether infiltrating mast cells could also increase PCa cells invasion *via* increasing the stem/progenitor cell population. We first found co-culturing with HMC-1 cells could induce PCa (LNCaP, C4-2 and C4-2B) cells to express more stem/progenitor cell markers including CD133, Nanog and CXCR4 (Figure 5A). We then applied flow cytometry to analyze the percentage of CD133^+^ stem/progenitor cells population in C4-2 cells, and results showed co-culturing with HMC-1 cells could increase CD133^+^ stem/progenitor cells population in C4-2 cells (Figure 5B). The sphere formation assay also revealed that infiltrating HMC-1 cells could further increase the self-renewal capacity in the C4-2 CD133^+^ stem/progenitor cell population (Figure 5C).

As expected, knocking-down AR in C4-2 cells with AR-siRNA could also increase the CD133^+^stem/progenitor cell population (Figure 5D). Importantly, applying an interruption approach for blocking the AR function during the co-culture system showed that infiltrating HMC-1 cells no longer were able to significantly increase the CD133^+^ stem/progenitor cell population in PCa C4-2 cells (Figure 5D), suggesting infiltrating HMC-1 cells might function through down-regulation of AR to increase PCa CD133^+^ stem/progenitor cell population.

Interestingly, we also confirmed the expression of AR and MMP9 in PCa stem/progenitor cells population. As expected, the MMP9 expression was up-regulated (Figure S4A) but AR expression was down-regulated significantly in CD133^+^ cells compared to CD133^−^ cells (Figure S4B), suggesting low expression of AR could increase PCa invasion capabilities *via* increasing stem/progenitor population after ADT.

Together, results from 4 different assays, stem/progenitor cell markers (Figure 5A), cell sorting (Figure 5B), sphere formation (Figure 5C) and interruption assay (Figure 5D), all suggest that enhanced mast cell recruitment may also be able to function through modulation of AR signaling to alter CD133^+^ stem/progenitor cell population in PCa cells to increase PCa cells invasion.

### Mechanism dissection why infiltrating mast cells could suppress PCa AR expression

After confirming the AR down-stream signals (either *via* AR→MMP9 or *via* AR→CD133^+^ stem/progenitor cell population) play key roles for infiltrating mast cells to enhance PCa cell invasion, we decided to dissect the AR up-stream signals to see how infiltrating mast cells may influence the AR expression in this co-culture system.

**Figure 6:**
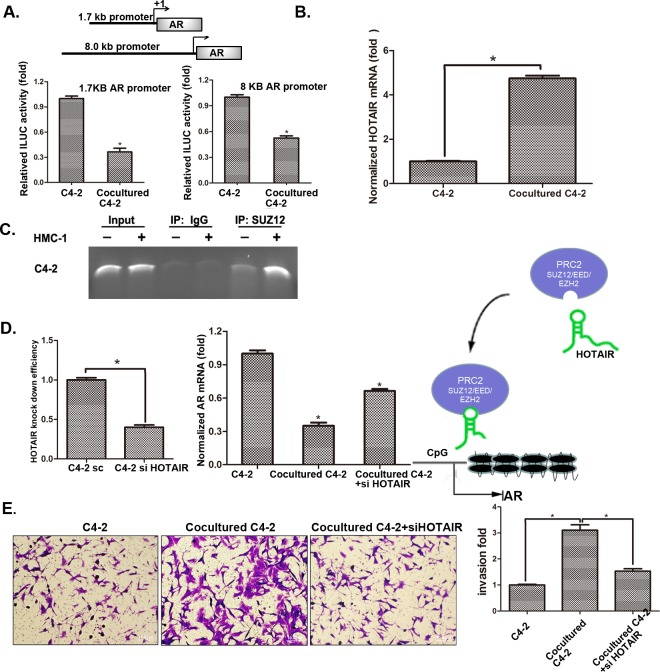
Mechanisms why mast cell recruitment suppressed PCa AR expression and increased PCa cells invasion **A**. QPCR shows HOTAIR mRNA level changes after co-culture of C4-2 cells with HMC-1 cells. **B**. AR promoter luciferase assay. We transfected C4-2 cells with two different AR promoter plasmids (1.7 kb and 8.0 kb) and then co-cultured with HMC-1 cells for 24 hrs and detected luciferase signal. **C**. ChIP assay results showed PRC2 complex could bind to the AR promoter. After 48 hrs co-culture, protein-DNA complex of C4-2 cells was cross-linked, sonicated, and precipitated by the SUZ12 antibody. The DNAs were purified from the pulldown complex and subjected to PCR. Rabbit IgG was used as the negative control. **D**. Knocking-down HOTAIR reversed AR expression. The left panel shows HOTAIR knock down efficiency after knocking down HOTAIR in C4-2 cells. We co-cultured with HMC-1 cells for 48 hrs, extracted RNA and detected AR mRNA level changes as shown in middle panel. Right panel Cartoon model of HOTAIR transporting the PRC2 complex to the AR promoter region. **E**. After knocking down HOTAIR, we co-cultured C4-3 cells with HMC-1 cells and detected C4-2 invasion ability changes. * p < 0.05.

We first demonstrated that infiltrating mast cells could decrease AR expression at the protein and mRNA levels (Figure 3). We then constructed luciferase reporter linked with AR promoter to confirm if infiltrating mast cells could also decrease AR expression at the transcriptional level. The results showed that infiltrating mast cells could decrease AR promoter transcriptional activity (Figure 6A). Importantly, after screening many candidate genes involved in the modulation of AR transcriptional activity, we found lnc-RNA HOTAIR in PCa cells could be increased after co-cultured with HMC-1 cells (Figure 6B and S5A). We also found the increased binding of lnc-RNA HOTAIR to PRC2 complex might then increase binding to the AR promoter in C4-2 and CWR22Rv1 cells after co-culture with HMC-1 cells (Figure 6C and S5B). Since the PRC2 complex is a silencing gene that requires HOTAIR for its binding to the target genes, we then applied HOTAIR-shRNA to suppress the PRC2 complex binding to the AR promoter. As shown in Figure 6D, adding HOTAIR-shRNA in C4-2/HMC-1 co-culture system decreased the PRC2 complex binding to the AR promoter (Figure S6) and further reversed the HMC-1 cells suppression of AR expression. As expected, with the HOTAIR-shRNA, the HMC-1 cells increased PCa C4-2 cell invasion was also partially reversed (Figure 6E).

Together, results from Figure 6A-E and S5-6 suggest that infiltrating HMC-1 cells may function through modulation of lnc-RNA HOTAIR-PRC2 complexes to suppress AR in PCa cells.

### Increased PCa metastasis with recruited mast cells in orthotopically xenografted mouse model

**Figure 7:**
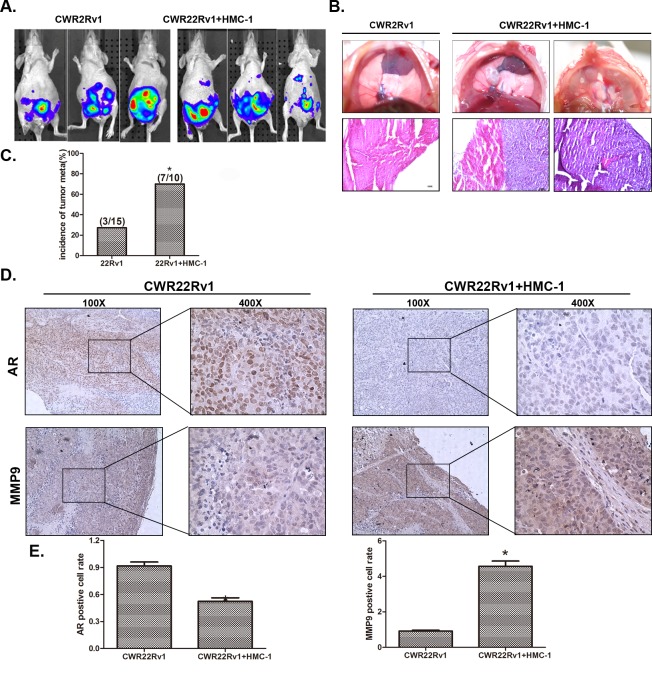
Mast cells promoted PCa invasion using *in vivo* orthotopic PCa model **A**. Orthotopic-implantation of mast cells together with PCa cells promotes PCa invasion. The CWR22Rv1 cells were transfected with pCDNA-luciferase (22rv-1-luc). CWR 22Rv1-Luc cells (1×10^6^) with or without mast cells (1×10^5^) were orthotopically implanted into the AP of nude mice. After 6 weeks implantation, the PCa growth was monitored by IVIS imaging following tail vein injection with Luciferin. **B**. Image illustrates metastasized tumors in the diaphragm. The top panels show the tumor mass in the diaphragm, and the bottom panels are the HE staining to confirm the tumors are cancer. **C**. Quantification data for tumor metastases in mice. **D**. IHC staining for AR and MMP9 in orthotopic mice tumor tissues. **E**. Quantification data for IHC staining. * p < 0.05.

To demonstrate the above *in vitro* cell results in the *in vivo* animal models, we then used a mouse model with orthotopically xenografted PCa CWR22Rv1 cells that were stably transfected with pCDNA-luciferase plasmid to monitor the PCa progression with IVIS Imaging system. After orthotopically xenografting 1×10^6^ CWR22Rv1 cells with or without 1×10^5^ HMC-1 cells for six weeks, we found (from IVIS image) increased metastatic foci in 6 out of 10 mice with xenografts of PCa and HMC-1 cells, compared to only 3 out of 15 mice having metastatic foci with CWR22Rv1 cells only (Figure 7A). We further identified one mouse to have metastatic foci in the diaphragm after sacrifice, suggesting 70% (7/10) of mice xenografted with co-cultured PCa and mast cells have metastatic foci *vs.* 20% (3/15) of mice xenografted with PCa cells alone (Figure 7C). The metastasis foci were also confirmed by staining PSA as compared with local tumor (Figure S7).

H&E staining in those metastatic foci confirmed those are PCa foci, including the one in diaphragm (Figure 7B). Importantly, IHC staining of AR and MMP9 expressions are also in agreement with *in vitro* co-culture studies showing infiltrated mast cells could decrease AR expression and increase MMP9 expression (Figure 7D-E).

## DISCUSSION

The tumor microenvironment with selective inflammatory immune cells plays an important role in PCa progression [9]. Mast cells are one of these immune cells that could be recruited into various tumor tissues to impact the tumor progression *via* influencing the angiogenesis [27]. Recent reports also found the infiltrated mast cells in the peri-tumoral compartment could be a novel independent prognostic marker for PCa, and it may also be linked to the progression into the castration resistant stage [12]. Our finding that recruited mast cells could enhance PCa growth and invasion provides strong *in vitro* and *in vivo* evidence to support mast cells roles in the late PCa castration resistant stage.

Mechanism dissection found recruited mast cells might function through modulation of AR signaling to enhance PCa cell invasion, which is in agreement with early studies showing targeting AR with AR-siRNA might lead to enhance PCa cell invasion *via* either TGF-β1/Smad3/MMP9 signaling [25] or STAT3-CCL2/CCR2 signaling [8]. Importantly, we are able to link the lnc-RNA HOTAIR-PRC2 signaling as an upstream signal to modulate AR at the transcriptional level. Early studies indicated that methylation of the AR promoter CpG islands might also be able to suppress the AR expression at the transcriptional level in PCa cells [28, 29]. PRC2, with H3K27 histone methyl transferase HMTase EZH2 and core components SUZ12 and EED, could influence the chromatin compaction [21] and lncRNA-HOTAIR (HOX antisense intergenic RNA) might interact with PRC2 for gene-silencing with a mechanism involving the methylation regulation [23]. With data showing mast cells could increase lncRNA HOTAIR to promote the binding of PRC2 complex (*via* SUZ12) to AR promoter CpG region for the suppression of AR expression, the axis of lnc-RNA-HOTAIR to→AT promoter methylation→reduced AR expression→increased PCa metastasis may represent a key mechanism to control the PCa metastasis at the later castration resistant stage.

Results from a Phase II study of abiraterone acetate treatment for castration resistant prostate cancer (CRPC) patients found that 79% of patients have a decline in PSA level of 50% or more, but 52% of PCa patients have either increased new bone lesions or intensity of existing bone lesions [30]. Cooperberg et al also reported the increased PCa specific mortality in clinical patients after ADT [31]. The recently published reports also found increased expressions of the epithelial-mesenchymal transition (EMT) marker following ADT [32], and Niu et al found that TRAMP mice with knocked-down AR in prostate epithelial cells also had increased metastatic PCa with mice dying earlier than the wild type TRAMP mice [33]. Finally, Lin et al demonstrated that ADT with the anti-androgens casodex or enzalutamide could increase the PCa cell invasion *via* either altering the TGFβ-1/Smad3/MMP9 signals [25] or influencing the infiltrating macrophages *via* reduced AR-mediated PIAS3 expression and then enhanced pSTAT3-CCL2 signals (32). Here we found another mechanism to explain why PCa cells treated with ADT using casodex or enzalutamide could have increased cell invasion *via* alteration of the recruitment of mast cells. These new findings not only strengthen the early conclusion that ADT with casodex or enzalutamide could increase PCa cell invasion and may also provide a potential new therapeutic approach *via* interrupting the infiltrating mast cells and their induced lncRNA-HOTAIR-PRC2 complex→AR→MMP9 signals.

In summary, our results concluded that infiltrating mast cells could increase PCa metastasis *via* modulation of lncRNA-HOTAIR-PRC2 complex→AR→MMP9 signals, which may provide us a potential therapeutic approach to better battle PCa metastasis *via* targeting this newly identified signaling from infiltrated mast cells.

## MATERIALS AND METHODS

### Cell culture

LNCaP and CWR22Rv1 cell lines were purchased from the American Type Culture Collection (ATCC, Manassas, VA). C4-2 and C4-2B cell lines were a gift from Dr. Jer-Tsong Hsieh of University of Southwestern Medical Center. Human mast cell line HMC-1 was a gift from Dr. John Frelinger of Eye Institute of University of Rochester Medical Center. HMC-1 was cultured in Iscove's modified Dulbecco's medium (IMDM) supplemented with 10% heat inactivated fetal bovine serum (FBS), 2 mM L-glutamine,100 IU/mL penicillin, and 50 μg/mL streptomycin. When indicated, HMC-1 cells were exposed to the differentiation reagent PMA (Sigma-Aldrich, Paso Robles, CA) at 20 ng/ml for 3 days. For the remainder of this study, PMA treated HMC-1 cells will be considered mature (differentiated) and untreated HMC-1 cells will be considered immature (undifferentiated) [34].

### Reagents and materials

GAPDH (6c5), β-actin (I-19), and AR (N-20) antibodies were purchased from Santa Cruz Biotechnology (Paso Robles, CA). MMP9 (ab38898) was from Abcam (San Diego, CA). SUZ12 (3737) was from Cell Signaling (Boston, MA). Human IL6 protein was from PROSPEC Corp (Houston, TX). IL6 and IL8 neutralizing antibody was from R&D (Minneapolis, MN). Crystal violet was from Fisher Scientific Company (Grand Island, NY). Anti-mouse/rabbit secondary antibody for Western Blot was from Invitrogen (Grand Island, NY).

### Mast cell recruitment assay

Mast cell migration was detected using a 24-well transwell assay. Briefly, PCa cells were placed in the lower chambers of the 24-well transwells. Mast cells (1×10^5^ cells/mL) were then seeded in the upper chambers. The upper and lower chambers were separated by an 8 μm polycarbonate filter coated with fibronectin (10 μg/ml, sc-29011 Santa Cruz, Paso Robles, CA) and dried for 1 hr in the hood. The chambers were incubated for 4 hours at 37°C. Filters were then scraped, washed, fixed with cold methanol, and stained with 1% toludine blue. Cell migration was measured by counting the number of cells attached to the lower surface of the filter. Each experiment was tested in triplicate. The results were expressed as the mean ± SEM of the number of migrating cells.

### Cell invasion assay

24-well (8 μm pores) transwell plates (Corning, Lowell, MA) were used for invasion assays. For *in vitro* invasion assays, the upper chambers of the transwells (8 μm) were pre-coated with diluted matrigel (BD Biosciences, Sparks, MD). Before performing invasion assays, PCa cells were cultured with either mast cells or CM for 48 hrs, and then wash out mast cells with PBS 3 times and then collected the co-cultured cells for further experiments. In brief, 10^5^ PCa cells (in serum free media) and 10% serum containing media were plated in the upper chambers. After 48 hrs incubation, invaded cells were stained with 0.1% crystal violet, and positively stained cells were counted. The cell numbers were calculated from counting six random fields. Quantitation indicates mean of triplicate repeats ± SEM.

### 3D invasion assay

The PCa cells invasion were also assayed by using 3D matrigel assay. In brief, we thawed Matrigel on ice and add 40 μl to each well of 8-well glass chamber slide (at 50 μl/cm2) and spread the Matrigel evenly, placed the slides in the cell culture incubator and allowed the Matrigel to solidify. We then plated 10^4^ PCa cells into each well with CM containing 5% Matrigel and 10 ng/ml EGF and changed media every 4 days. PCa cells take about 7 days to form acini-like structures. The number of structures in each of 10 different random fields under 100× microscope were counted.

### RNA extraction and quantitative real-time PCR analysis

Total RNAs were isolated using Trizol reagent (Invitrogen). 1 μg of total RNA was subjected to reverse transcription using Superscript III transcriptase (Invitrogen). Quantitative real-time PCR (qRT-PCR) was conducted using a Bio-Rad CFX96 system with SYBR green to determine the mRNA expression level of a gene of interest. Expression levels were normalized to the expression of GAPDH RNA.

### Western blot analysis

Cells were lysed in RIPA buffer and proteins (20 μg) were separated on 10-12 % SDS/PAGE gel and then transferred onto PVDF membranes (Millipore, Billerica, MA). After blocking membranes, they were incubated with appropriate dilutions (1:1000) of specific primary antibodies. The blots were incubated with HRP-conjugated secondary antibodies and visualized using ECL system (Thermo Fisher Scientific, Rochester, NY).

### Luciferase reporter assays

Human AR promoter regions of 1.8kb and 7kb were amplified and cloned into the pGL3 vectors as we described previously [35], and obtained as a gift from Dr. Yan Dong of Tulane University. The luciferase reporter assays were performed according to the methods used in a previous study [36]. PCa cells were lysed and the luciferase activity was detected by the dual luciferase assay using pRL-TK-luciferase as the internal control. Each sample was normalized by pRL-TK-luciferase activity, and data were presented as mean ± SEM from at least three independent experiments.

### Chromatin immunoprecipitation assay (CHIP)

CHIP assay was performed as previously reported [37, 38]. In brief, cell lysates were precleared sequentially with normal rabbit IgG (sc-2027, Santa Cruz Biotechnology) and protein A-agarose. Anti-SUZ12 antibody (2.0 μg) was added to the cell lysates and incubated at 4°C overnight. For the negative control, IgG was used in the reaction. Specific primer sets designed to amplify a target sequence within human AR promoter were: hAR: Forward, 5′- CGACAGCCAACGCCTCTTG-3′; Reverse, 5′- CCTTGCTTCCTCCGAGTCTTTAG-3′; PCR products were identified by agarose gel electrophoresis.

### *In vivo* metastasis studies

Male 6- to 8-week old nude mice were used. CWR22Rv1 cells were engineered to express luciferase reporter gene (PCDNA3.0-luciferase) by stable transfection and the positive stable clones were selected and expanded in culture. 15 mice were injected with PCa cells (1 × 10^6^ of luciferase expressing cells, as a mixture with Matrigel, 1:1) and other 10 mice were co-injected with PCa cells co-cultured with (HMC-1) (1 × 10^5^) to anterior prostate (AP). Metastasis in live mice was monitored by using a Fluorescent Imager (IVIS Spectrum, Caliper Life Sciences, Hopkinton, MA), following tail vein injection of Luciferin, at 6 different time points (3, 4, 5, and 6 wks after injection). After a final monitoring with the Imager, mice were sacrificed and the metastases were further examined by H&E. All animal studies were performed under the supervision and guidelines of the University of Rochester Medical Center Animal Care and Use Committee.

### H&E staining

The tissue sections were de-waxed and rehydrated routinely. The sections were stained in hematoxylin for 5 min, and washed in running tap water for 5 min. Then the sections were stained in eosin for 30 sec, dehydrated, and mounted by routine methods. The representative fields were chosen to present in the figures.

### Histology and IHC staining

Mouse prostate tissues were fixed in 10% (v/v) formaldehyde in PBS, embedded in paraffin, and cut into 5 μm sections. Prostate sections were deparaffinized in xylene solution and rehydrated using gradient ethanol concentrations, and immunostaining was performed.

### Statistics

All statistical analyses were carried out with SPSS 16.0 (SPSS Inc, Chicago, IL). The data values were presented as the mean ± SEM. Differences in mean values between two groups were analyzed by two-tailed Student's *t* test. *p* < 0.05 was considered statistically significant.

## SUPPLEMENTARY MATERIAL, FIGURES AND TABLE


