# Correction: Infiltrating mast cells enhance prostate cancer invasion via altering LncRNA-HOTAIR/PRC2-androgen receptor (AR)-MMP9 signals and increased stem/progenitor cell population

**DOI:** 10.18632/oncotarget.13912

**Published:** 2016-12-12

**Authors:** Lei Li, Qiang Dang, Hongjun Xie, Zhao Yang, Dalin He, Liang Liang, Wenbing Song, Shuyuan Yeh, Chawnshang Chang

**Present**: Due to an error made during the assembly of Figure 2B and S2. It has come to the authors’ attention that wrong figure panels in Figure 2B and S2 were provided. After checking the question figure, we found that, by mistake, during preparation of these multiple set of figure panels, some of the images that had been included were incorrect.

**Correct**: Correct Figure 2B and S2 is provided below. The authors sincerely apologize for this error.

Original article: Oncotarget. 2015; 6(16):14179-90. doi: 10.18632/oncotarget.3651.

**Figure 2:**
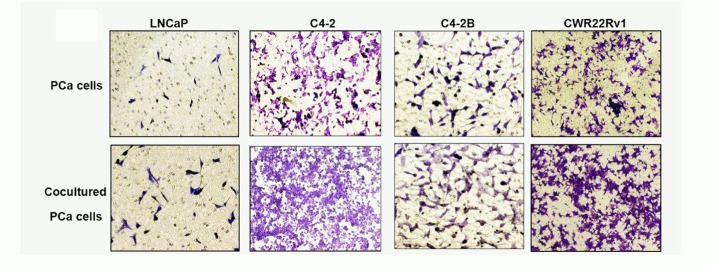
Increased infiltrating mast cells to PCa enhanced PCa cell invasion B. Images show mast cells co-cultured PCa cells have a higher invasiveness. The top panels show untreated PCa cells as control, the bottom panels show PCa cells co-cultured with HMC-1 cells.

**Figure S2:**
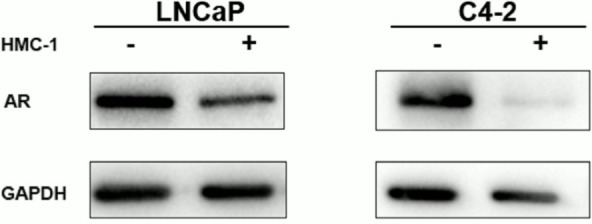
Co-culturing mast cells with PCa cell conditioned media (CM) inhibited PCa cell LNCaP and C4-2 cells AR expression

