# Supplementary material for: Infiltrating mast cells enhance prostate cancer invasion via altering LncRNA-HOTAIR/PRC2-androgen receptor (AR)-MMP9 signals and increased stem/progenitor cell population
Source: Oncotarget. 2015 Mar 26;6(16):14179–90. doi: 10.18632/oncotarget.3651 (PMC4546459; doi:10.18632/oncotarget.3651)
Supplement: Supplementary file 1 [file oncotarget-06-14179-s001.pdf]

Infiltrating mast cells enhance prostate cancer invasion via altering LncRNA-HOTAIR/PRC2-androgen receptor (AR)-MMP9 signals and increased stem/progenitor cell population

Supplementary Material

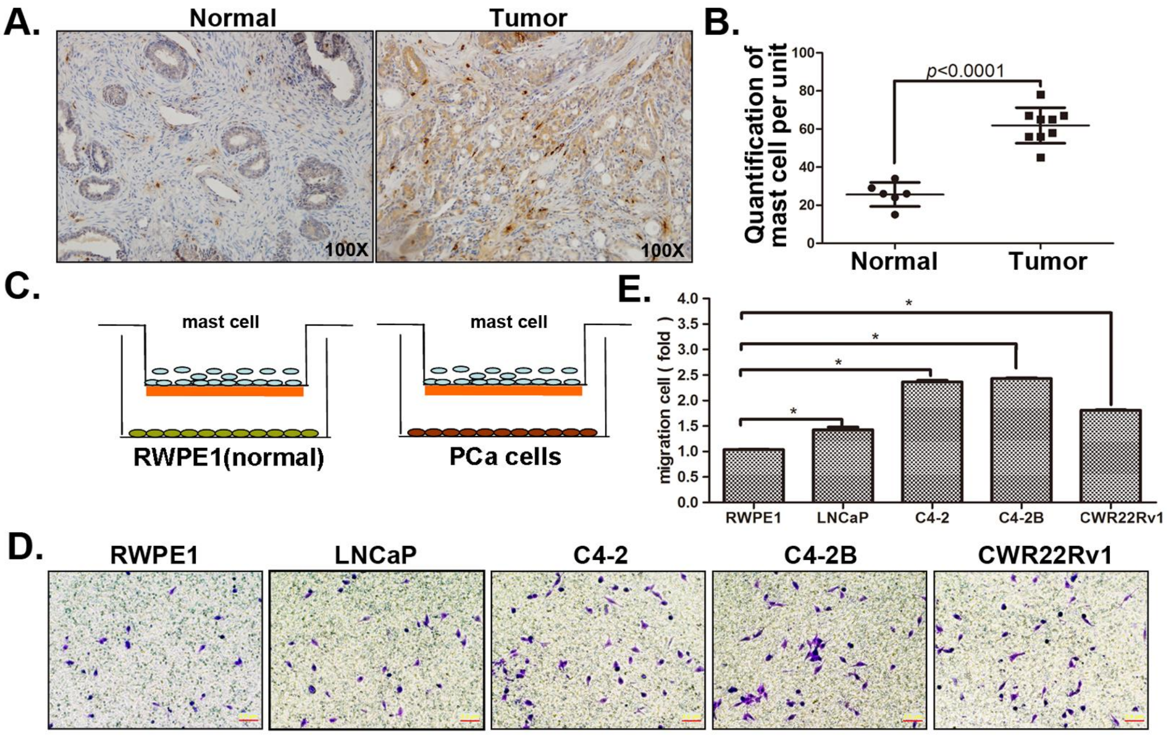

Figure S1: Prostate cancer (PCa) tissues recruit more mast cells than normal prostate tissues. A. IHC staining of anti-trypsatase antibody to show mast cell staining in human PCa and normal tissues. B. Quantification of mast cell numbers in PCa tissues and normal prostate tissues. C. Cartoon illustration of the mast cell migration assay. The insert upper wells were pre-coated with 10 ng/ml fibronectin. HMC-1 cells (mast cells, 1×10^5) were placed in the upper chamber and the PCa cells were placed in the bottom wells to assay the migration of HMC-1 cells. After 4 hrs, the bottom sides of insert wells were fixed and stained to visualize the migrated HMC-1 cells. D. PCa cells promote HMC-1 cells migration. HMC-1 cells (1×10^5) were added in the upper well, and we seeded non-malignant prostate RWPE1 cells and 4 different PCa cell lines, LNCaP, C4-2, C4-2B and CWR22Rv1 (1×10^6) in the lower wells to do migration assay for 4 hrs. E. Quantitation data for migrated HMC-1 cells. Results were presented as mean±SD. Statistical analysis was done by two-tailed Student's t test, * $p < 0.05$.

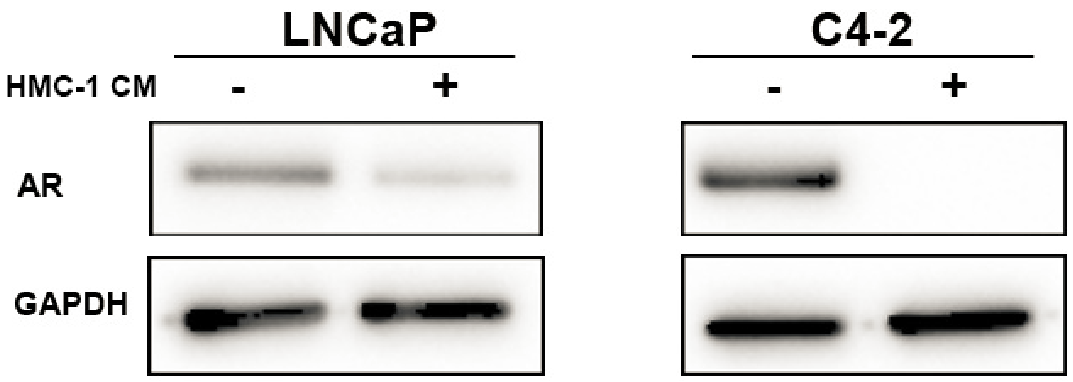

Figure S2: Co-culturing mast cells with PCa cell conditioned media (CM) inhibited PCa cell LNCaP and C4-2 cells AR expression.

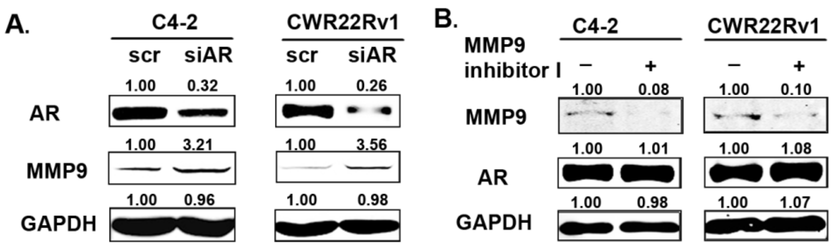

Figure S3: AR regulates MMP9 expression in PCa cells. A. MMP9 expression was detected by Western blot after knocking down AR in C4-2 and CWR22Rv1 cells. B. AR and MMP9 expression after treating PCa cells with 10 μg/ml MMP9 inhibitor I.

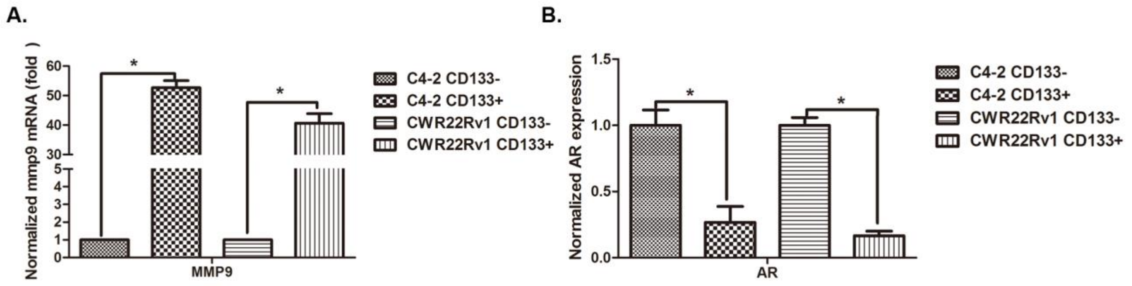

Figure S4: MMP9 and AR expression in CD133+ stem/progenitor cells. We isolated $CD133^+$ stem/progenitor cells by FACS, and then assayed MMP9 and AR expression in $CD133^+$ and $CD133^-$ cell populations by QPCR. **A**. MMP9 expression in $CD133^+$ and $CD133^-$ populations in C4-2 and CWR22Rv1 cells. **B.** AR expression in $CD133^+$ and $CD133^-$ populations in C4-2 and CWR22RV1 cells. * $p < 0.05$.

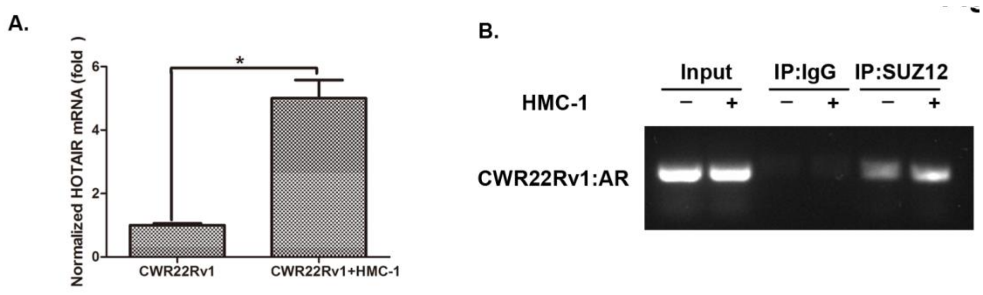

Figure S5: Infiltrated mast cell could increase HOTAIR expression and lead SUZ12 complex to bind on the promoter of AR in CWR22Rv1 cells. A. QPCR shows HOTAIR mRNA level changes after co-culture with HMC-1 cells in CWR22Rv1 cells. B. PRC2 complex CHIP assay. After co-culture for 48 hrs, we used ultrasonic dispersion DNA, pulled down by SUZ12 antibody, collected and purified bound DNA and processed PCR. Normal rabbit IgG was used as control. * $p < 0.05$.

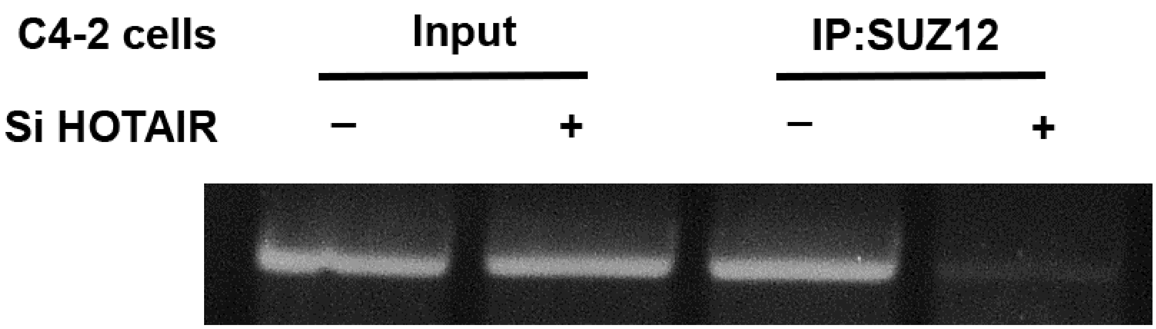

Figure S6: Knocking down HOTAIR expression and decreasing of SUZ12 complex to bind on the promoter of AR in co-cultured C4-2 cells. PRC2 complex CHIP assay. After C4-2 cell co-culture with HMC-1 cells for 48 hrs, the HMC-1 cells were washed out and C4-2 cells were collected. We use ultrasonic dispersion DNA, pulled down by SUZ12 antibody, collected and purified bound DNA and processed PCR. * $p < 0.05$.

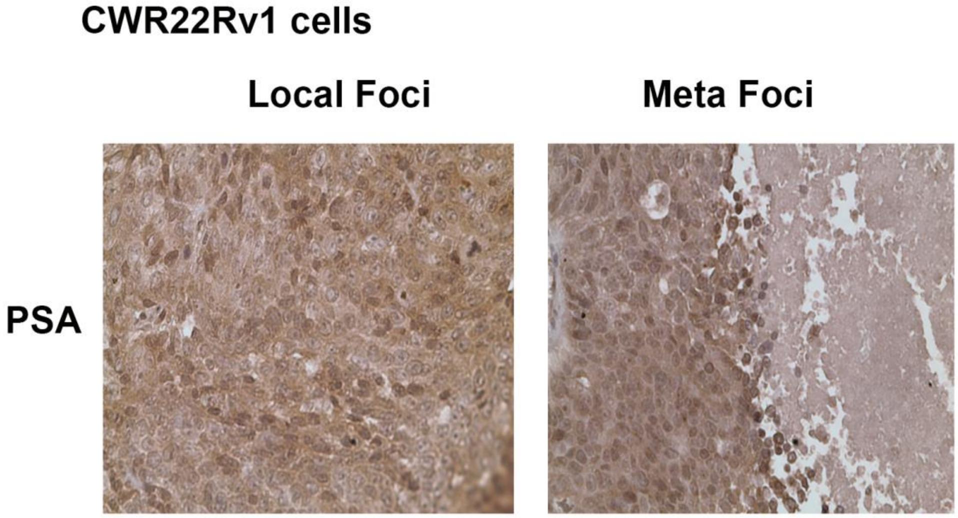

Figure S7: Identification of the metastasis foci in orthotopic PCa model. IHC staining for PSA in metastatic foci tissues.

Supplementary Table 1: The invasion and metastasis-related genes in PCa cells using Q-PCR array. The invasion and metastasis-related genes were assayed in HMC-1 co-cultured PCa cells compared with control PCa cells.

Metastsis related gene					
Beta-catenir	CD44	COL4A2	CTSL1	DAB2IP	EGF
EPHB2	EZH2	FLT4	HGF	HIF1a	HIF2a
HPSE	HRAS	IGF1	JMJDIA	KAI1	KISSIA
MET	METAP2	MMP1	MMP2	MMP9	MMP13
MTA1	MTIMMP	MYC	NEDD9	NME4	SERPINB
SMAD3	SRC	SYK	TGFB1	TIMP1	TIMP2
TM4SF1	TP53	VEGFa	ZEB1		
